# Is the detection of accelerated sea level rise imminent?

**DOI:** 10.1038/srep31245

**Published:** 2016-08-10

**Authors:** J. T. Fasullo, R. S. Nerem, B. Hamlington

**Affiliations:** 1 National Center for Atmospheric Research, Boulder, CO, 80305, USA; 2 University of Colorado, Boulder, CO, 80309, USA; 3 Old Dominion University, Norfolk, VA, 23529, USA

## Abstract

Global mean sea level rise estimated from satellite altimetry provides a strong constraint on climate variability and change and is expected to accelerate as the rates of both ocean warming and cryospheric mass loss increase over time. In stark contrast to this expectation however, current altimeter products show the rate of sea level rise to have decreased from the first to second decades of the altimeter era. Here, a combined analysis of altimeter data and specially designed climate model simulations shows the 1991 eruption of Mt Pinatubo to likely have masked the acceleration that would have otherwise occurred. This masking arose largely from a recovery in ocean heat content through the mid to late 1990 s subsequent to major heat content reductions in the years following the eruption. A consequence of this finding is that barring another major volcanic eruption, a detectable acceleration is likely to emerge from the noise of internal climate variability in the coming decade.

Driven by both the warming of the oceans and mass loss of the cryosphere, global mean sea level (GMSL) is among the most powerful indicators of a changing climate^1^. Originally estimated from a network of tide gauges and restricted to coastlines, it has been derived from satellite measurements over the ice-free oceans since shortly after the launch of the TOPEX/Poseidon satellite in late 1992 with unprecedented accuracy and stability^2^. During a given year, the height of the global seas can now be estimated to within a few millimeters revealing a mean rise of just over 3 mm yr^−1^ 2,3. However the holistic nature of sea level has also led to challenges in its interpretation and it can be unclear at times whether its variations arise from changes in ocean heating, cryospheric melting, or the amount of water stored over land. Disentangling these various influences remains an ongoing science objective^1^,^4^,^5^.

Among the major unanswered questions is why GMSL acceleration has not yet been detected in the altimeter record, given the increasing rates at which glacial and ice sheet melt are estimated to have occurred^6^,^7^ and as greenhouse gas concentrations have risen^8^. The answer to this question has considerable policy relevance and debates over whether such an expected acceleration should be considered at the local policy level have at times been contentious^9^. GMSL also has the potential to serve as an early indicator of accelerated climate change as it is less sensitive to the large internal variability that characterizes the surface temperature record, as evidenced by the recent so-called hiatus in global warming^10^, [Fig. S3]. Prevailing hypotheses for the lack of observed acceleration have maintained that changes in water storage over land^11^ or even drift in the satellite instruments may be to blame^12^. Here it is demonstrated that the environment in which the era began was itself highly anomalous due to the preceding eruption of Mt Pinatubo on June 15, 1991, which cooled the oceans and decreased water storage over land and in the atmosphere. The net effect of these changes was to lower sea level prior to the altimeter era and induce an anomalous rise in the era’s early years. It is proposed that this early anomalous rise masked the acceleration that would have otherwise occurred in the broader record. A consequence of this interpretation is that as the altimeter record lengthens, and in the coming decade barring another major volcanic eruption, accelerated rise will likely be detected.

Since late 1992 until the present, the TOPEX/Poseidon (T/P), Jason-1, and Jason-2 satellite altimeter missions have continuously measured sea level changes between ±66° latitude with a temporal resolution of about 10 days, as shown in Fig. 1. With their precision orbit determination, dual-frequency measurements (to remove ionosphere delays) and microwave radiometer (to remove delays due to water vapor in the troposphere), these missions have created an unrivaled 23-year climate data record of sea level change that is now being extended with the successful launch and deployment of the Jason-3 satellite earlier this year. Great care has been taken in calibrating these measurements via overlaps between missions and comparison to tide gauge sea level data^2^,^13^. When averaged globally, the record provides an estimate of GMSL with a seasonal mean accuracy of 1–2 mm^2^. Over the 23-year time series, it shows that GMSL has been rising at a rate of 3.3 ± 0.4 mm yr^−1^, but with notable inter-decadal variability. Our current best estimate of the rates during the first (1993–2002) and second (2003–2012) decades of the altimeter era are 3.5 and 2.7 mm yr^−1^, respectively, though important sources of uncertainty persist and raise caution regarding the record’s early years [dashed line, Fig. 1; also Fig. S1]. There are several theories to explain this variability^11^,^12^, but here we present an additional explanation, with important implications for anticipated near-future acceleration.

## Results

### Insight from Tide Gauges

Prior to satellite altimetry, the primary source of sea level data is from historical tide gauge records. These data provide a significantly longer time series relative to that of satellite altimetry, with a few records extending back into the 18^th^ century. Several studies have estimated GMSL from the tide gauge record using a variety of techniques^14^,^15^,^16^,^17^,^18^,^19^,^20^. The resulting estimates of the GMSL trend from 1900 to 1990 range from 1.2 mm yr^−1^ to 1.9 mm yr^−1^, albeit with significant decadal variability about this long-term trend [1, Fig. 13.7b]. Coupled with the higher GMSL trend observed during the satellite altimeter record discussed above, the tide gauge record demonstrates unequivocal acceleration since the early 1900 s, with estimates ranging from 0.009 +/− 0.002 mm yr^−2^ 17 to 0.017 +/− 0.003 mm yr^−2 ^20. Based on these same studies, however, the majority of the acceleration arises from a shift that occurs around 1990 when the rate of sea level rise increases to the satellite-measured trend of 3.3 mm yr^−1^.

While the tide gauge record may provide a ballpark estimate for what to expect during the altimeter era, it provides only weak quantitative information regarding what acceleration should be expected. In practice, calculating GMSL from 1900 to the present is a challenging problem based on the spatial and temporal sampling characteristics of available gauges. There is little consensus across tide gauge studies on the rate and acceleration of GMSL over the past century, thus making it difficult to interpret the altimeter era in a broader context. As an alternative approach to understanding sea level variability, we therefore seek to estimate and remove effects that obscure a possible underlying acceleration from the altimeter record itself. By doing so, we can potentially estimate the acceleration in GMSL directly from altimetry.

### The GMSL Influence of the 1991 Mt Pinatubo Eruption

On June 15, 1991 the second largest volcanic eruption of the twentieth century in terms of aerosol radiative forcing began on the Philippine island of Luzon^21^,^22^. Estimates of the amount of ash deposited in the stratosphere ranged from 20 to 30 Tg^21^ (approximately equal to a tenth the mass of all mankind) inducing a cooling of the globe and especially the world’s oceans. Model-based estimates of the eruption’s cooling effects suggest that the recovery of ocean heat content during the 1990’s may have increased sea level rise by as much as 0.5 mm yr^−1^ on average in the decade following the eruption^23^,^24^,^25^,^26^,^27^. The precise temporal evolution of the increase, and the impacts of the eruption on terrestrial and atmospheric water reservoirs remain largely unknown however; as quantifying these impacts is complicated by contemporaneous climate variability that can mask or amplify the response^28^. Moreover, as many of the tools now in place for monitoring climate, such as for example the ARGO network of ocean temperature sensors^29^ and the GRACE satellite for estimating terrestrial water storage^30^, were yet to be deployed at the time of the eruption, quantifying its precise climatic effects is nontrivial. However, recent specially designed climate model experiments now provide additional insight. What they reveal is that the eruption had a profound and temporally complex influence on several contributors to sea level and especially the amount of heat stored in the oceans, even when compared to background variations in climate, with major consequences for perceptions of GMSL acceleration during the altimeter era.

**Figure 2:**
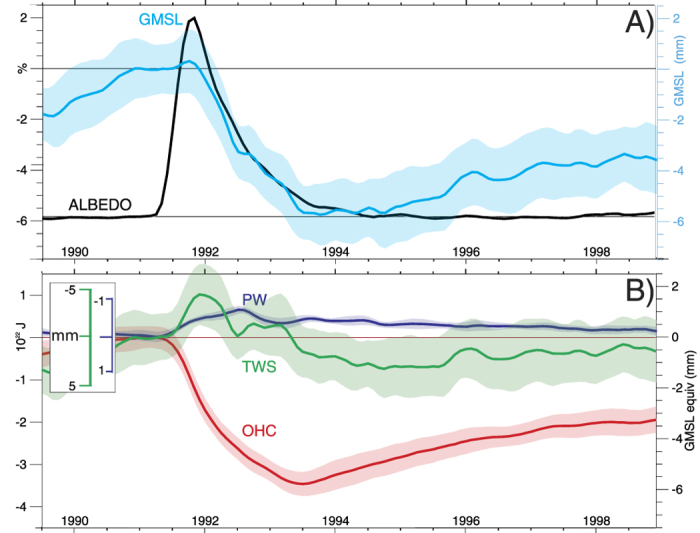
Simulated sea level rise contributions during and following the eruption of Mt Pinatubo. Shown are changes in (**A**) clear-sky albedo over the tropical oceans (30 N – 30°S) as an indicator of the eruption’s radiative effects and associated global mean sea level (GMSL) anomalies. In (**B**) contributions from ocean heat content (OHC) atmospheric water vapor (PW) and terrestrial water storage (TWS) estimated from the LE are shown. The large standard deviation across ensemble members (shaded) highlights the obscuring effect of natural climate variability on the eruption’s influence in observations.

The CESM Large Ensemble (LE) is a 40-member ensemble of state-of-the-art coupled climate simulations spanning the 20^th^ and 21^st^ centuries using estimated historical forcings including volcanic eruptions [see Methods for a detailed description]. The simulated responses of GMSL and its individual contributors to the 1991 eruption of Mt Pinatubo are summarized in Fig. 2. Immediately following the eruption, aerosols in the stratosphere blocked sunlight and cooled the surface. Surface temperatures quickly dropped, particularly over land due to its relative lack of thermal inertia^22^, [Fig. S2]. In turn, the atmosphere cooled, reducing the amount of moisture stored within it as water vapor. A cooler surface evaporated less moisture and was less convectively unstable, leading to a subsequent reduction in rainfall globally and disproportionately over land where diminished land water storage and runoff were a consequence of the eruption^31^.

As these terrestrial and atmospheric changes are associated with reductions in their storage of water, their initial influence was to delay by about six months the eruption’s main effect on sea level, which was a significant and rapid drop arising from a reduction in ocean heat content (OHC). The short timescale of the terrestrial and atmospheric influences relative to the oceans however limited their persistence, and by the beginning of the altimeter era in 1993, a GMSL drop of 5 to 7 mm from the eruption is estimated to have occurred, due largely to cooling of the oceans. While the LE’s estimated OHC deficit is difficult to verify directly, given the large uncertainties and errors inherent in global ocean observations^32^, confidence in the simulated response is bolstered by satellite estimates of the Earth’s radiative imbalance, which strongly constrain the magnitude of ocean cooling and agree closely with simulated fluxes [Fig. S3]. Confidence in the ability of the LE to capture fundamental features of the eruption is therefore high.

## Discussion

This assessment of the sea level budget during Mt Pinatubo’s 1991 eruption and in the several years thereafter has far reaching implications. First, it suggests that our monitoring of sea level via altimetry began in a highly anomalous environment, one in which OHC had been significantly depressed by the eruption while the offsetting influences of the atmosphere and land surface had largely diminished. As the oceans equilibrated from Pinatubo’s initial cooling, sea level experienced an anomalously rapid rise. It is therefore suggested that the GMSL rise estimated from the past decade is likely to be more representative of the background rate due to climate change than that observed during altimetry’s initial decade. The eruption of Pinatubo is therefore also reaffirmed as a contributor to the apparently large shift in the rates of rise between the gauge and altimeter eras, consistent with the findings of previous studies^23^,^24^,^25^,^26^,^27^.

The budgets simulated by the LE also have implications for the estimation of acceleration. From them, it is estimated that the rate of rise from 1993–2002 is subject to anomalous contribution of about 5 to 7 mm from the recovery to Pinatubo’s oceanic cooling (Fig. S2)^23^,^24^,^25^,^26^,^27^. This contribution therefore likely eclipses the background acceleration inferred from the tide gauge record. Given however that no such major eruption has occurred since 1991, a reasonable expectation is also that an accelerated rate of rise may emerge in the near future, particularly as the influences of climate variability and instrument drift reported in previous studies abate^11^,^12^.

**Figure 3:**
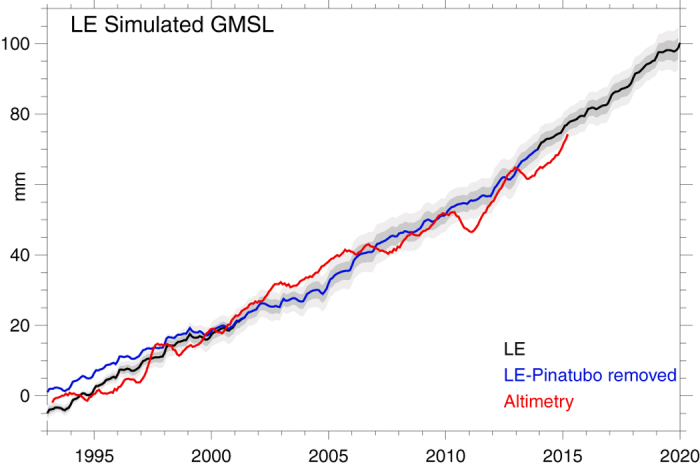
Sea level rise associated with ocean heat storage and the sum of all contributions estimated from LE budgets and cryospheric contributions (see Methods). Shaded are 1-σ (light) and 2-σ ranges of internal variability. The mean LE estimate with Pinatubo effects removed is also shown (blue) along with the altimeter record (red).

**Figure 4:**
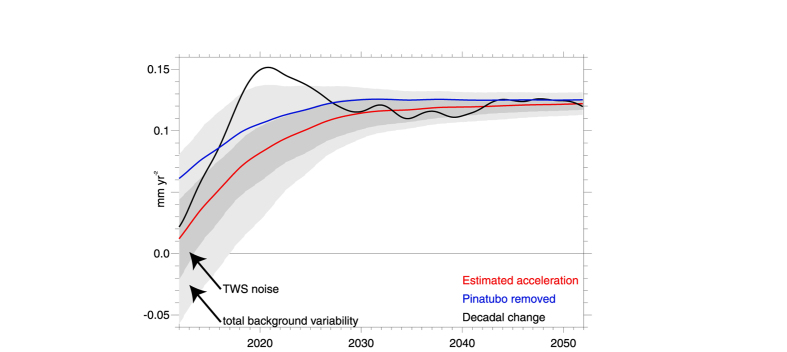
Estimated acceleration in sea level rise based on the budget from the LE and cryospheric contributions using trend differences 1) between the first and second half of a record beginning in 1993 and ending in a given year (abscissa, red) and 2) for the trailing two decades, based on given end years (abscissa, black). Acceleration with Pinatubo effects removed is also shown (blue). The 2σ spread in simulated acceleration estimates is shown for all contributions (light grey) and the contribution due to TWS alone (dark grey).

An estimate of this near-future GMSL acceleration can be made using LE projections of OHC, TWS, and PW in conjunction with independent estimates of future ice sheet losses [1, Figs 3 and 4]. The estimate suggests that, as discussed above, it is unsurprising that acceleration has yet to be detected given the forced response to Pinatubo and the noise of internal climate variability in both OHC and TWS (shaded regions of Figs 2, 3, 4), and potential retrieval biases^12^. Moreover, the result also demonstrates that as anthropogenic influences continue to increase (as a result of both increasing greenhouse gas concentrations and decreasing anthropogenic aerosol emissions), a detectable acceleration of GMSL rise is likely to emerge as it exceeds the noise of background climate variability (shaded) in the coming years. The main contributor to this acceleration is the accelerated increase in OHC, which is offset somewhat by increasing but secondary influences from atmospheric and terrestrial storage (Figs. S2 and S4), while a key component of the noise obscuring acceleration is the variability of TWS. Moreover, when the effects of the Mt Pinatubo eruption estimated form the LE are removed (blue), acceleration becomes apparent, even in the present day. The magnitude of the acceleration in the mid-21^st^ century is estimated here to be 0.12 mm yr^−2^, though this value depends strongly on future ice sheet losses, which are highly uncertain^1^. Its accurate estimation depends both on the accuracy of altimeter retrievals and our ability to distinguish it from internal variability, which can be pronounced in some years but over the long term becomes increasingly negligible, particularly if acceleration is estimated from the full post-1993 record (red line in Fig. 4).

With the launch of Jason-3 earlier this year, it is reasonable to ask what new climate insights the instrument may bring. This analysis concludes that if the lifespan of the instrument is comparable to that of its predecessor, Jason-2, the acceleration suggested in earlier studies^8^,^16^,^33^ will likely emerge from the noise of internal climate variability during its lifetime, barring another major volcanic eruption. Moreover, given the unique strengths of sea level as a stable and holistic measure of climate change, the broader altimeter record is likely to stand as one of the benchmark measures of accelerating changes in the climate system.

## Methods

The problem of detecting the emergence of sea level acceleration is fundamentally one of resolving signal (GMSL’s forced response) from noise (internal variability). To estimate both the noise and the signal, the CESM1-CAM5 Large Ensemble (LE) is used. The LE a 40-member ensemble of coupled climate model simulations spanning 1920 to 2100 34. The simulations incorporate historical and 21^st^ century forcings estimated based on CMIP5 protocols^35^,^36^. While the CESM1-CAM5 ocean component conserves ocean volume by design, terms contributing to sea level rise can be estimated from energy and water budgets. Relevant terms include terrestrial water storage (TWS), atmospheric total water (PW), and ocean heat content (OHC). Advances in simulating TWS in the CESM1-CAM5 are considerable relative to previous model generations^37^. Global mea level (GMSL) changes are computed based on the conversion of PW and TWS to GMSL using the relative ratio of ocean (0.72) and land (0.28) areas to the globe. GMSL contributions from OHC are estimated based on the conversion of 3•10^22^ J = 5 mm provided inRef. 38. Included in TWS is the local mass balance over the ice sheets but absent from such variability are changes in ice sheet dynamics, which are beyond the scope of current coupled climate models. Additional “all-but-one” simulations were performed using full historical forcings excluding only volcanic aerosols in order to isolate the perturbation arising from volcanic effects. Only 4 members of these simulations were available yet changes in the forcing in these runs are low frequency in nature and therefore a smaller ensemble size is found to be adequate for characterization of background non-volcanic forced variability. Smoothed fits to OHC, PW, and TWS are used for removing the effects of the 1991 Mt Pinatubo eruption. The TWS contribution to acceleration in Fig. 4 has been computed by generating sea level estimates using ensemble mean values for OHC and PW, rather than the values of individual members, and computing associated ranges of acceleration over time.

Ice sheet contributions to historical and future sea level rise are taken from IPCC AR5, Chapter 13, Figure 13.11 in which changes due to glaciers, Greenland, and Antarctica are estimated from their lower-bound estimates. The sensitivity of acceleration to other choices has also been explored, including middle and upper range estimates of loss, and found to further hasten GMSL acceleration. The choice of the lower bound of these contributions is found to provide a conservative estimate for near term GMSL acceleration.

## Additional Information

**How to cite this article**: Fasullo, J. T. *et al*. Is the detection of accelerated sea level rise imminent? *Sci. Rep.* **6**, 31245; doi: 10.1038/srep31245 (2016).

## Supplementary Material

Supplementary Information

## Figures and Tables

**Figure 1:**
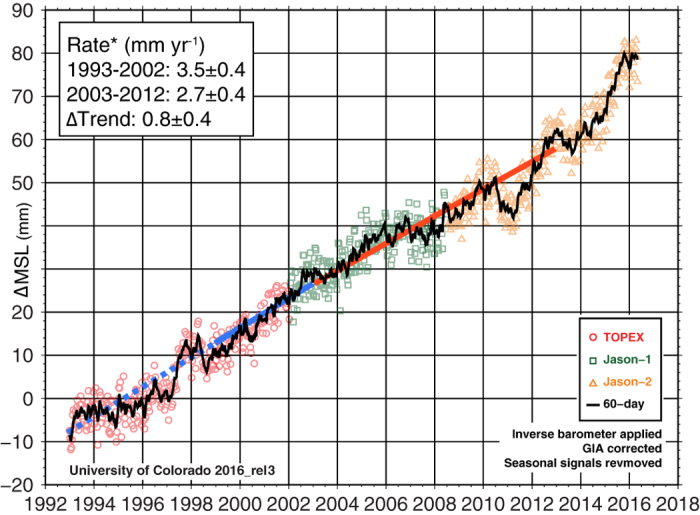
The altimeter record with decadal rates of change indicated. Estimates during the early stages of the record (dashed) are particularly subject to instrument related uncertainty (12).
