# Erratum: Is the detection of accelerated sea level rise imminent?

**DOI:** 10.1038/srep33622

**Published:** 2016-11-10

**Authors:** J. T. Fasullo, R. S. Nerem, B. Hamlington

Scientific Reports 6: Article number: 3124510.1038/srep31245; published online: 08 10 2016; updated: 11 10 2016

In the Supplementary Information file originally published with this Article, there was a typographical error in the legend of Figure S2 where “El Chichón” was incorrectly given as “El Chicón”. In addition, Figure S3 and its accompanying Figure legend were incorrect. The Reference ‘Church, John A. et al. Revisiting the Earth’s sea‐level and energy budgets from 1961 to 2008. Geophys. Res. Lett. 38, 18 (2011)’ was also omitted. These errors have been corrected in the Supplementary Information that now accompanies the Article.

