# Supplementary material for: Is the detection of accelerated sea level rise imminent?
Source: Sci Rep. 2016 Aug 10;6:31245. doi: 10.1038/srep31245 (PMC4978990; doi:10.1038/srep31245)
Supplement: Supplementary Information [file srep31245-s1.doc]

**Supplementary Material**

**Sources of Error in Trend Estimation During the Altimeter Record**


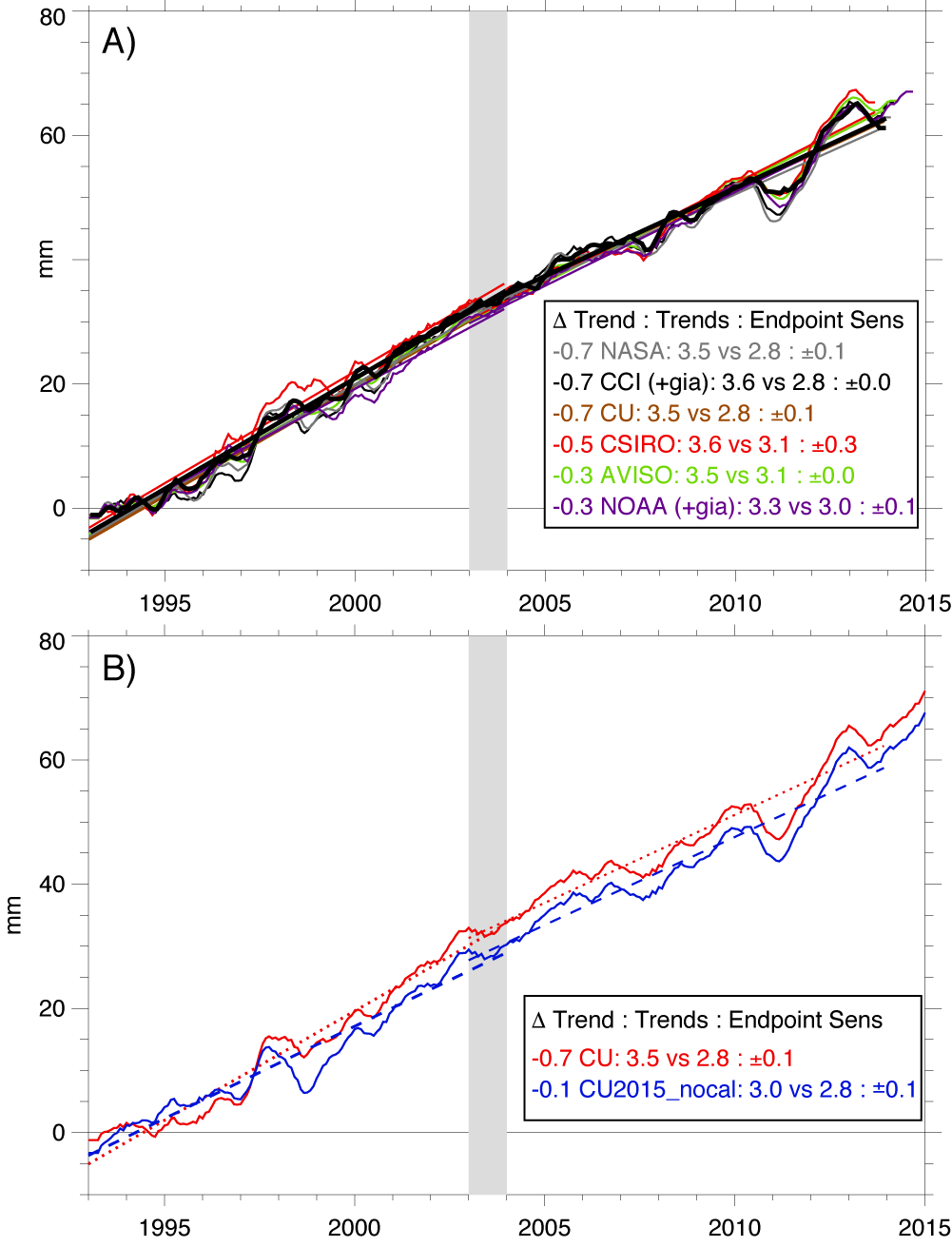


**Figure S1**: Decadal trends in the altimeter record and their sensitivity to A) reprocessing data center and chosen endpoints, and B) calibration with tide gauges.

The record of global mean sea level change from the TOPEX/Poseidon, Jason-1, and Jason-2 missions is well established [Nerem et al., 2010; Leuliette and Scharroo, 2010; Ablain et al., 2009], but characterizing the long-term errors in this time series remains a challenge. The current 23-year record shows a significantly higher rate of sea level rise over the first half of the record as compared to the last half of the record in the records provided by various reprocessing centers. The difference can be up to 0.7 mm yr^-1^ depending on processing adopted (Fig. S1a). Watson et al. [2015] recently used comparisons to tide gauges to determine that the TOPEX part of the record might have a GMSL trend that is biased high. This further suggests that the decadal difference in the rate of sea level rise over the entire 23-year record might be much smaller, which has implications for the determination of the accelerations of GMSL over this record such as when the calibration is removed (Fig. S1b, blue line). Research on these issues is ongoing and awaits a reprocessing of the TOPEX measurements, as well as a revalidation of the GMSL record using tide gauge measurements [e.g. Mitchum, 2000]. As a result, this paper does not present a definitive assessment of the acceleration of sea level rise during the altimeter era, but rather suggests a mechanism for the masking acceleration by non-anthropogenic sources, a mechanism that is relevant for the interpretation of the altimeter era irrespective of the optimal calibration of TOPEX.

**Sea Level as a Measure of Accelerated Climate Change**


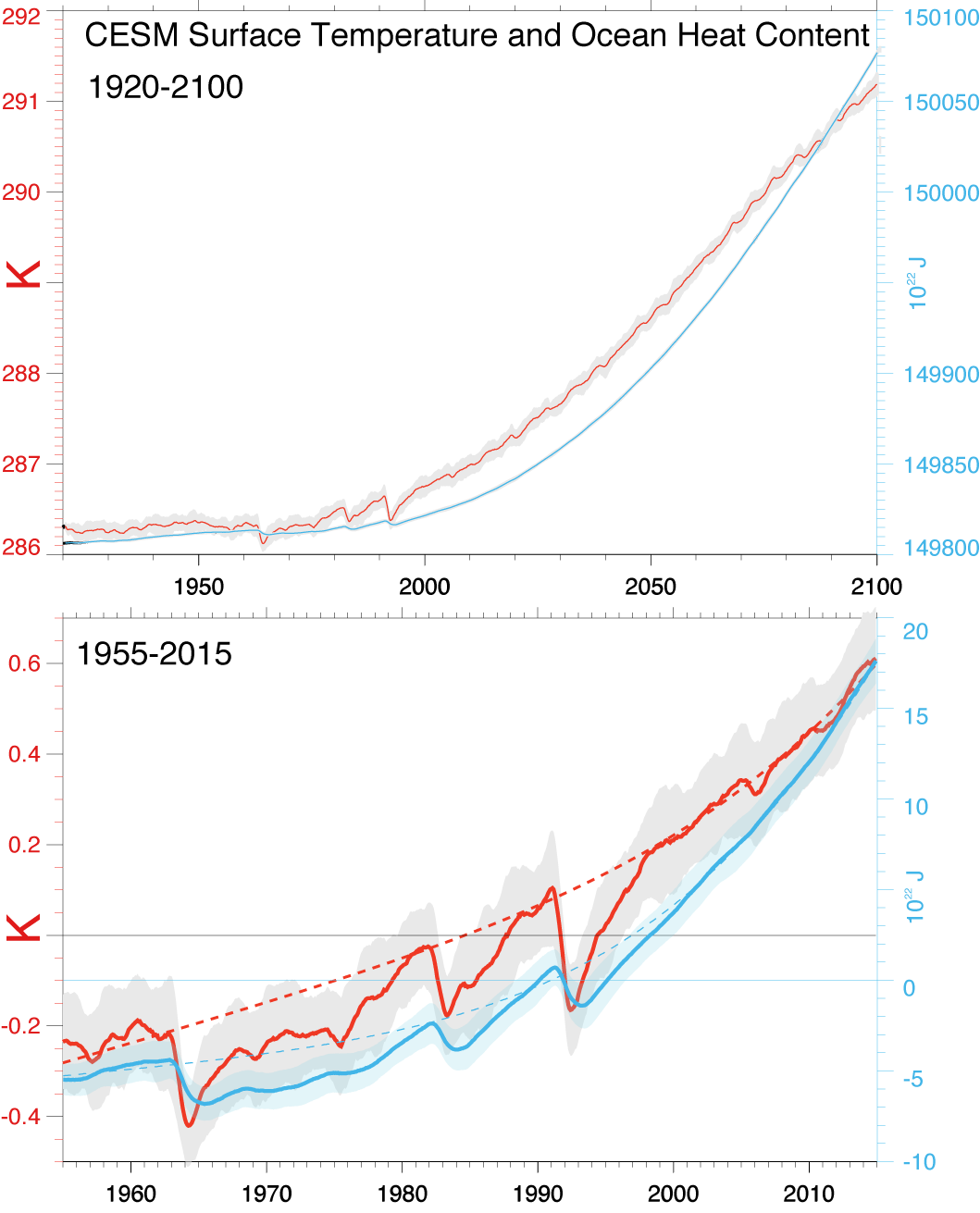


**Figure S2**: Simulated acceleration and 1-σ range of internal variability (shading) in surface temperature (red) and ocean heat content (blue), based on the LE. The forced responses without volcanic effects are also estimated (dashed).

One of the key strengths of GMSL as an index of climate change is its relative insensitivity to internal variability. Here this is demonstrated by showing the spread across ensemble members of the LE. In contrast to surface temperature, which exhibits large inter-member spread (red line, grey shading), GMSL (blue line and shading) exhibits small ensemble spread promoting the estimation of trends and acceleration in short records. The effects of volcanic eruptions on OHC and GMSL are longer than for temperature as evidenced by the lingering effects of the 1982 eruption of El Chicón are likely to have also influenced GMSL during the altimeter record and balance approximately the effects of Mt Pinatubo extending past 2002.

**Satellite Validation of the CESM1-CAM5 Large Ensemble**


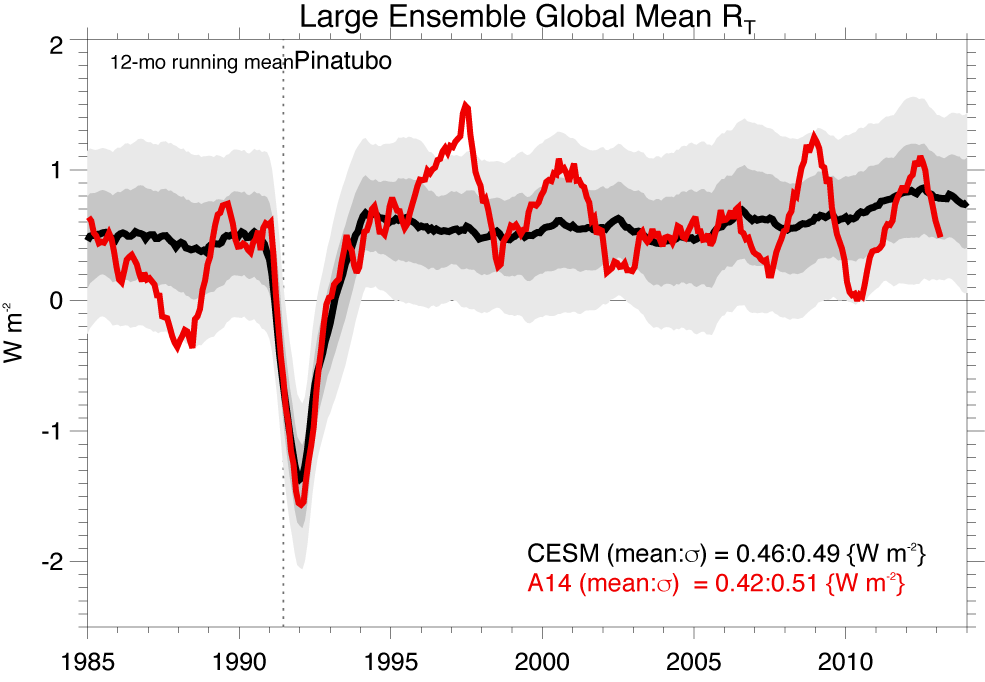


Using blended ERBS and CERES records in conjunction with other observations and models, Allen et al. 2014 constructed a top of atmosphere radiative budget extending from 1985 through 2013. The observed imbalance agrees closely with that of the LE with deviations from the one and two σ ranges of ensemble spread occurring only during major ENSO events and the exceptional 1997/98 El Niño event, respectively. Agreement of the radiative perturbation following the eruption (dotted line) is very strong and tightly constraints the simulated OHC response.


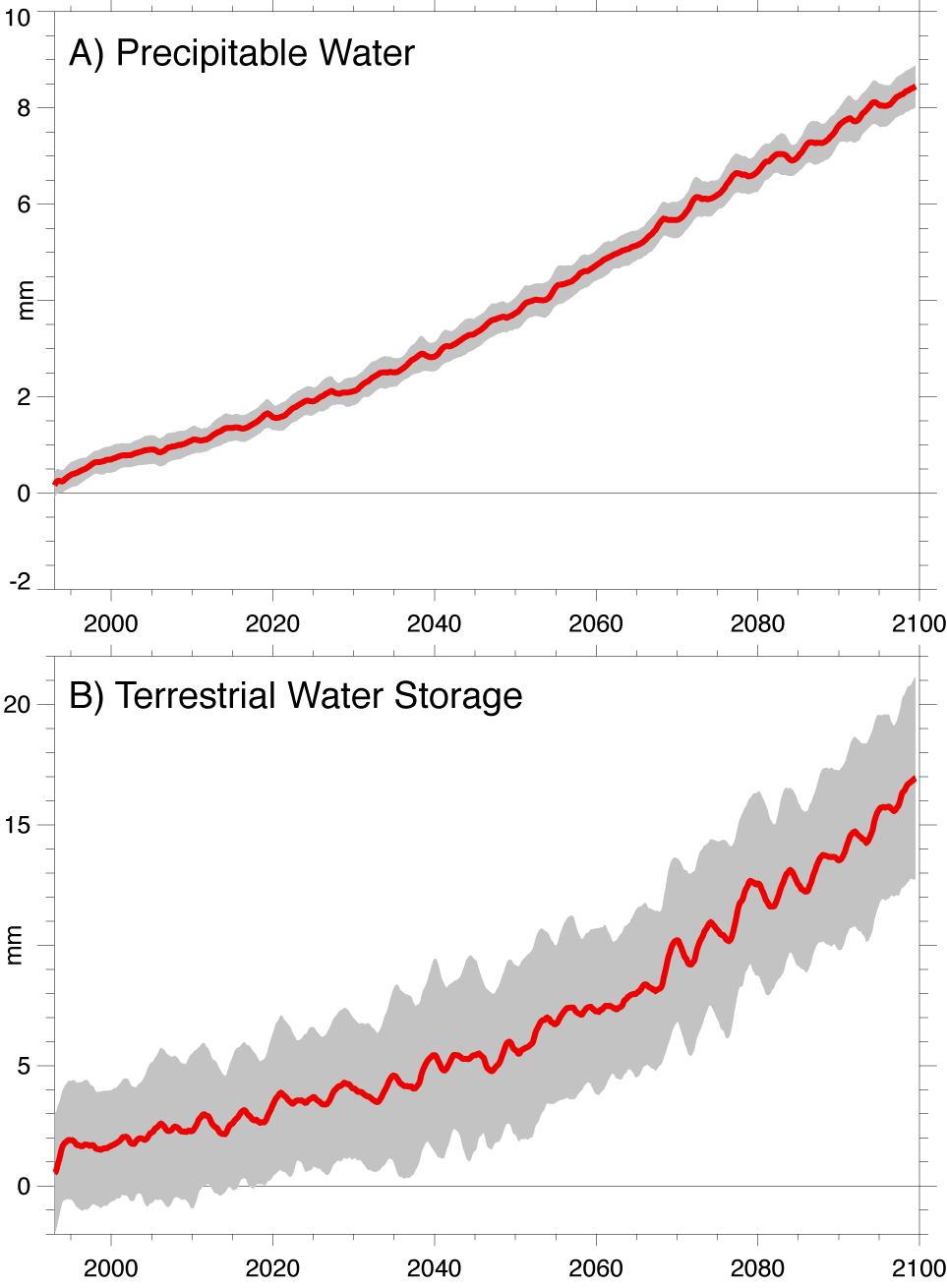


**Figure S4**: Projected increases in water storage in the atmosphere (PW, A) and on land (TWS, B) through the 21^st^ century are shown. Weak accelerations in increases in both reservoirs are apparent. Despite this however a net GMSL acceleration is simulated as a result of strong acceleration in OHC uptake (Fig. S2).

**References**

Allan, Richard P., et al. "Changes in global net radiative imbalance 1985–2012." *Geophysical Research Letters* 41.15 (2014): 5588-5597, doi: 10.1002/2014GL060962.

Mitchum, Gary T. 2000. “An Improved Calibration of Satellite Altimetric Heights Using Tide Gauge Sea Levels with Adjustment for Land Motion.” *Marine Geodesy* 23 (3): 145–66, doi:10.1080/01490410050128591.

Reager, J. T., et al. "A decade of sea level rise slowed by climate-driven hydrology." *Science* 351.6274 (2016): 699-703, doi: 10.1126/science.aad8386.
